# Predictability of short implants (< 10 mm) as a treatment option for the rehabilitation of atrophic maxillae. A systematic review

**DOI:** 10.4317/medoral.20949

**Published:** 2016-03-06

**Authors:** José-Luis Sierra-Sánchez, Fernando García-Sala-Bonmatí, Amparo Martínez-González, Carlos García-Dalmau, José-Félix Mañes-Ferrer, Alejandro Brotons-Oliver

**Affiliations:** 1 Associate Professor of the Master in Advanced Oral Implantology, European University of Valencia, Valencia, Spain

## Abstract

**Background:**

Short implants (< 10 mm) are one of the treatment options available in cases of limited vertical bone. A purpose of this paper is to evaluate the predictability of short implants as an alternative to technically molthough such implants are now widely used, there is controversy regarding their clinical reliability. There complex treatments in patients with atrophic maxillae, based on a systematic review of the literature and the analysis of the implant survival rates, changes in peri-implant bone level, and associated complications. It is postulated that short implants offer clinical results similar to those of longer implants.

**Material and Methods:**

A Medline-PubMed search was made covering the period between January 2004 and December 2014 (both included). Studies in English published in indexed journals, involving at least 20 implants and with a follow-up period of at least 12 months were considered. A manual search in four high impact journals was also conducted.

**Results:**

A total of 37 studies meeting the inclusion criteria were included in this review. 9792 implants placed in over 5000 patients were analyzed.

**Conclusions:**

Based on the results of this review, short implants are seen to offer clinical results in terms of survival, bone loss and complications similar to those of longer implants.

**Key words:**Survival rate, clinical results, dental implants, oral implants, short implants, short lengt

## Introduction

Bone resorption occurring after tooth loss in either the upper maxilla or the mandible can give rise to an atrophic alveolar crest. In most such cases, a functional and esthetically satisfactory dental implant supported rehabilitation is not possible. According to Araujo & Lindhe (1), tooth loss gives rise to physiological resorption of the alveolar process. This resorption is characterized by a decrease in both the number of trabeculae and in bone density, as well as loss of bone width and height. Depending on the time elapsed and the location within the maxillae, resorption will affect alveolar bone to one extent or other. It has been well established that bone loss in the first year after tooth loss is much greater than the loss observed over the subsequent years.

In the upper maxilla, bone resorption characteristically occurs towards the midline. This circumstance, added to the pneumatization of the maxillary sinus, can make implant placement in the posterior region more complicated. In the anterior region of the mandible, bone resorption occurs from the buccal plate towards the lingual aspect, while in the posterior areas it usually occurs from the lingual towards the buccal aspect. This fact gives rise to a centrifugal resorption pattern, which is characteristic of the mandible.

The posterior regions of both maxillae usually present less available bone height, as a consequence of bone resorption. In the upper maxilla the main anatomical limitation is caused by the pneumatization of the maxillary sinus, while in the mandible the mandibular canal is the structure that conditions the available bone height. For this reason, posterior regions of both maxillae are good candidates for rehabilitation using short dental implants.

Several surgical techniques have been described for the rehabilitation of patients with maxillary and mandibular atrophy using dental implants. These techniques originally attempted to increase the amount and quality of available bone, based on guided bone regeneration procedures, sinus lift techniques, block grafts and alveolar bone distraction.

Although all these techniques offer good results, they can be considered technically demanding procedures that in many cases give rise to complications such as graft failure, wound infection, a worse postoperative course, increased morbidity, longer treatment times, and higher economic costs for the patient. As an alternative to these techniques, the placement of short dental implants has been proposed for the rehabilitation of atrophic alveolar crests. According to Das Neves *et al.* (2), short implants are defined as implants measuring less than 10 mm in length. Other authors consider short implants to be implants measuring 8 mm or less in length - implants measuring 10 mm being regarded as conventional implants, due to their widespread use in recent years.

Some previous publications have found these short implants to offer clinical results comparable to those obtained with longer implants – the implant survival rates ranging between 92.3% according to Slotte *et al.* (3) and 100% as published by Anitua *et al.* (4) in the posterior region of the mandible, and between 94.6% according to Renouard & Nisand (5) and 100% as published by Taschieri *et al.* (6) in the posterior region of the upper maxilla.

Other factors to bear in mind when considering the use of short implants are their design and surface characteristics. In this regard, a rough surface means that despite the reduced implant length, the effective bone-implant contact surface area would be increased when being compared to a smooth surface.

Some three-dimensional finite element studies previously published have suggested that stress distribution is greater at a crestal level. According to these studies, the first three or four implant threads support most of the load. Therefore, maximum bone tension is independent of implant length - implant diameter being regarded as a more determinant factor than implant length.

When rehabilitating patients with missing teeth, one of the parameters to be taken into account is the influence of the crown-implant ratio upon the viability of the rehabilitation (in relation with biomechanics and stress distribution). When using short implants, the prognosis might be regarded as poorer as a result of the development of peri-implant bone loss. However, in 2009 Blanes (7) reported no relationship between crown-implant ratio and peri-implant bone loss.

Regarding the prosthetic rehabilitation of these implants, there is some controversy as to whether splinting should be used in all cases or not. According to Bahat (8), 60% of the failed short implants (< 7 mm) were single implants. This study points to prosthetic splinting as one of the main factors conditioning implant survival in the case of posterior regions rehabilitation procedures.

- Purpose 

The aim of the present study is to evaluate the predictability of short implants as an alternative to technically more demanding treatments, based on a systematic review of the literature and the analysis of the implant survival rates, changes in peri-implant bone level, and complications associated to the use of dental implants under 10 mm in length.

## Material and Methods

- Search strategy

A Medline-PubMed search was made of studies published in English and covering the period between January 2004 and December 2014 (both included). The key words used in the search included a combination of the following terms: “survival rate”, “clinical results”, “dental implants”, “oral implants”, “short implants”, “short length”. The Boolean operators “AND” and “OR” were used. In order to minimize electronic search bias, a manual search was made for relevant articles in the following high impact journals: “The International Journal of Oral and Maxillofacial Implants”, “Clinical - Oral Implants

**Figure 1:**
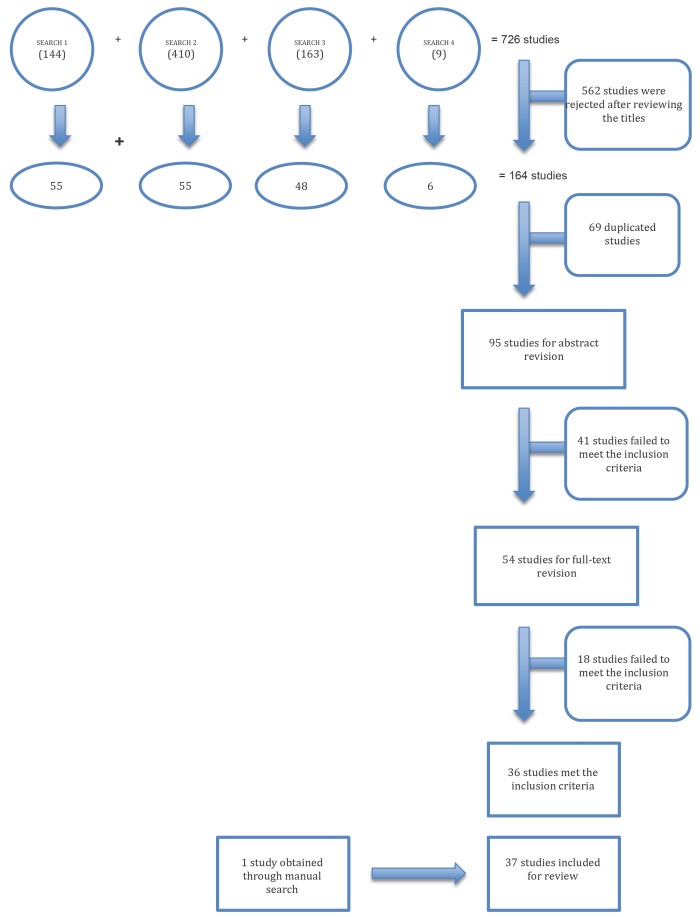
Study screening and inclusion criteria.

Research”, “Journal of Periodontology”, “Clinical Implant Dentistry and Related Research” and “European Journal of Oral Implantology”( Fig. 1).

- Study screening and inclusion criteria.

Two reviewers carried out the search. The variables of interest were implant survival, changes in peri-implant bone level, and associated complications. Implant survival was defined as implant persistence in the mouth at the time of evaluation.

The studies included in the review were required to meet the following criteria:

- Full-text articles in English, published in indexed journals between January 2004 and December 2014 (both included).

- Presentation of clinical results with implants measuring < 10 mm in length (no additional bone regeneration techniques to gain bone height allowed).

- Randomized clinical trials and clinical cohort studies of a prospective or retrospective nature conducted in humans, and involving a minimum of 20 implants.

- A follow-up period of at least 12 months.

In a first phase, two reviewers independently assessed titles and abstracts for relevance, and then debated upon them. A third reviewer was consulted in order to clear up any possible discrepancies. In a second phase, the full text of the selected articles meeting the inclusion criteria was subjected to additional analysis by two reviewers.

- Data extraction

All of the included studies were reviewed and analyzed independently. The variables related to the study design were extracted (year of publication, type of study and follow-up, number of patients, number of implants, mean age of the patients, inclusion or exclusion of smokers, and type of opposing dentition), along with the characteristics of treatment (implant surface, implant length and diameter, treated maxilla and localization of the implants, type of connection, characteristics of the surgical technique, type of prosthetic restoration, insertion torque and bone quality). The variables associated to treatment outcome (survival rate, peri-implant bone loss and associated complications) were also analyzed.

## Results

Figure 1 shows the results of the electronic and manual searches. Out of a total of 54 reviewed full-text articles, 36 met the inclusion criteria and were selected. One further article was added from the manual search.

The following variables were studied in the 37 finally included articles:

1. Variables associated to study design ( Table 1).

**Table 1:**
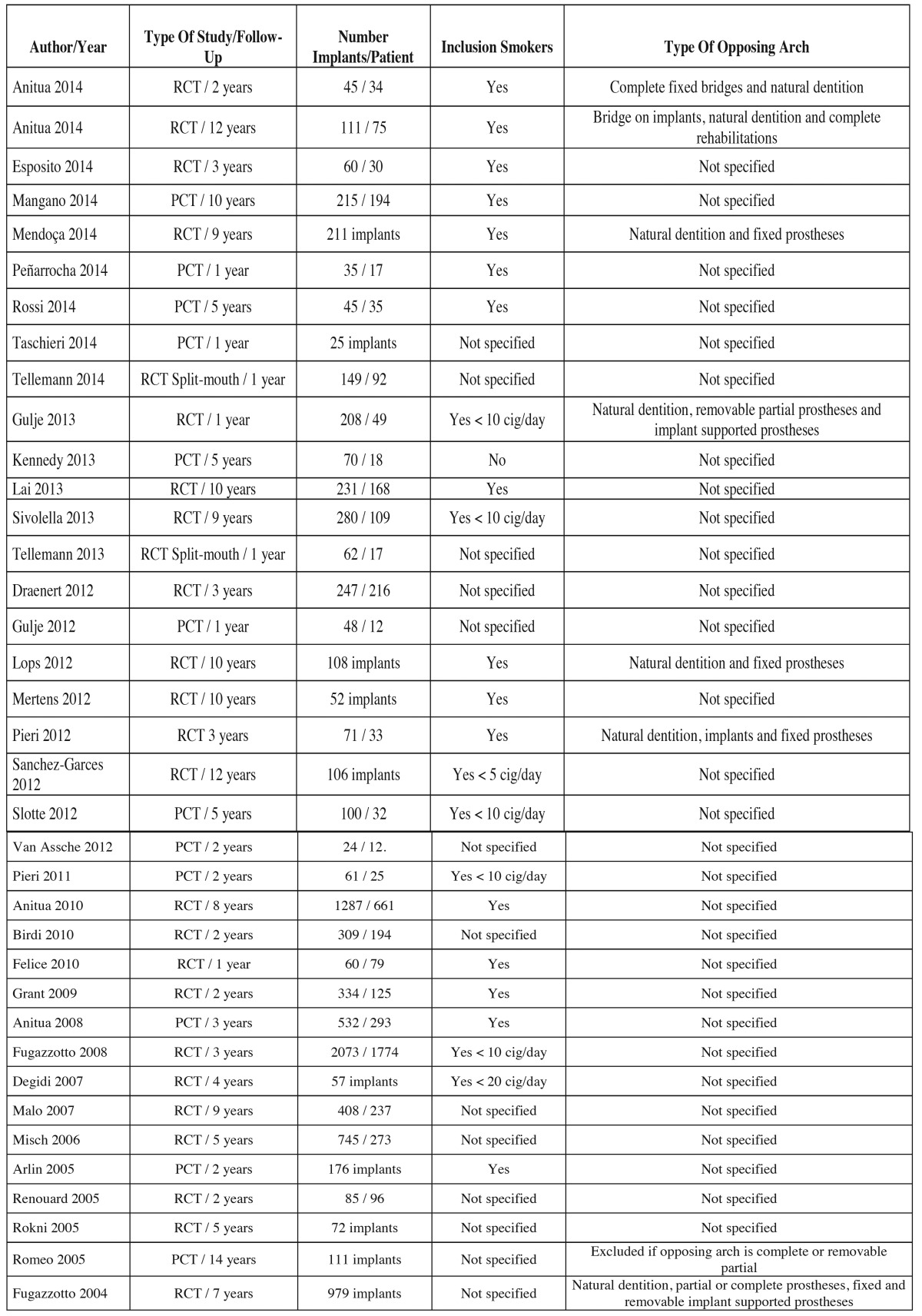
Variables associated to study design.

The review included a total of 37 studies published between 2004 and 2014. Of these, only 6 were randomized clinical trials. We also included 12 prospective and 19 retrospective cohort studies. The follow-up period of the studies ranged from 12 months to 14 years in the article published by Romeo *et al.* (9).

The 37 studies included over 5000 patients. Twelve studies involved more than 100 patients. The mean patient age ranged from 45.9 years to 62.1 years. In this review a total of 9792 implants were included.

Twenty-three of the 37 studies included smokers. Most of these articles established a limitation of between 5-10 cigarettes per day. Only one study published excluded smokers entirely, while 13 studies failed to indicate whether smokers were included or not. Twenty-nine of the reviewed studies provided no information on the type of opposing dentition. Seven studies specified the presence of natural teeth or fixed dentures (both teeth or implant supported) in the opposing arch, while only three publications published removable dentures (partial or complete) in the opposing arch.

2. Variables associated to treatment characteristics ( Table 2 and  Table 2continue continue).

**Table 2:**
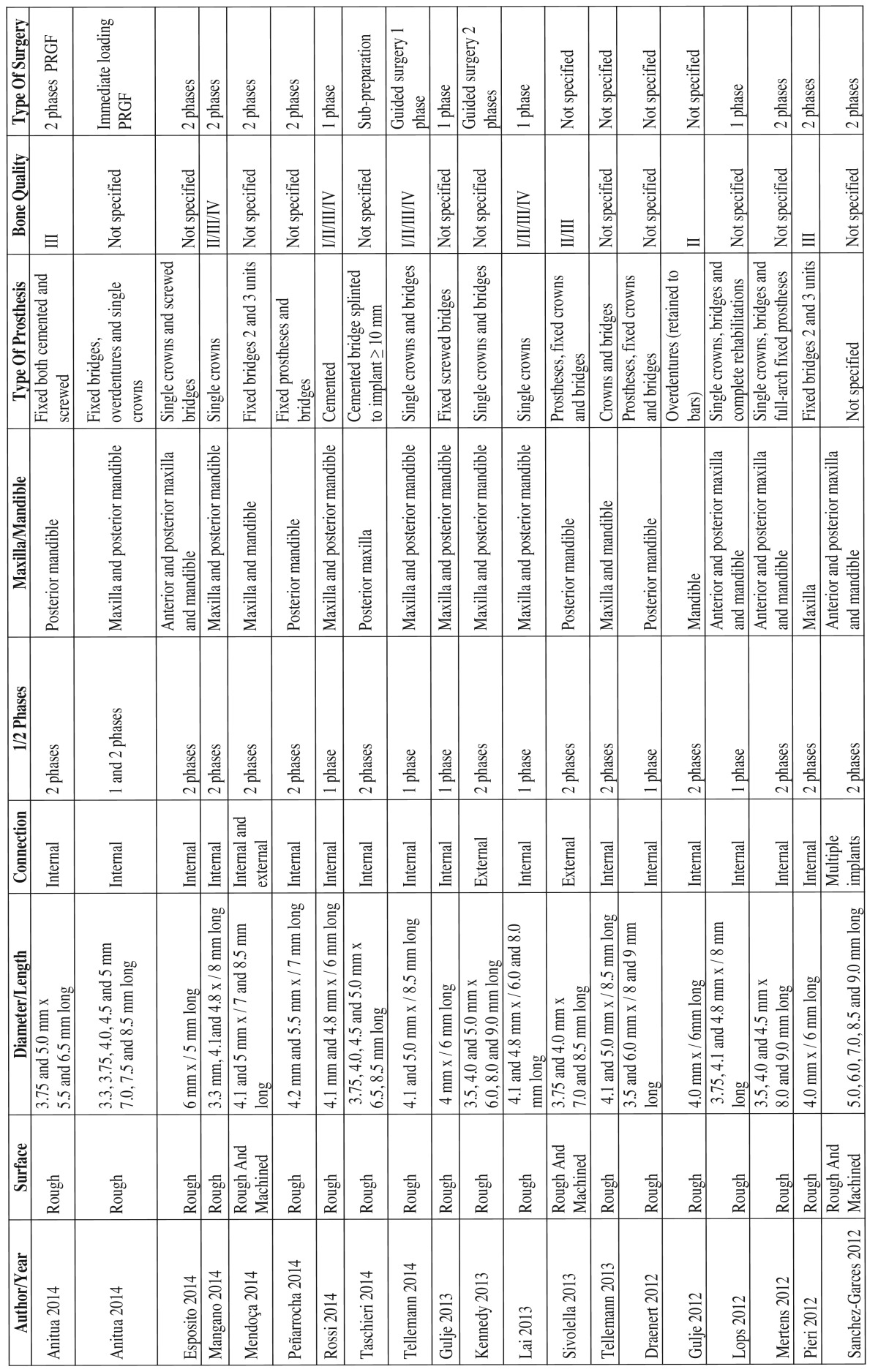
Variables associated to treatment characteristics.

**Table 2continue:**
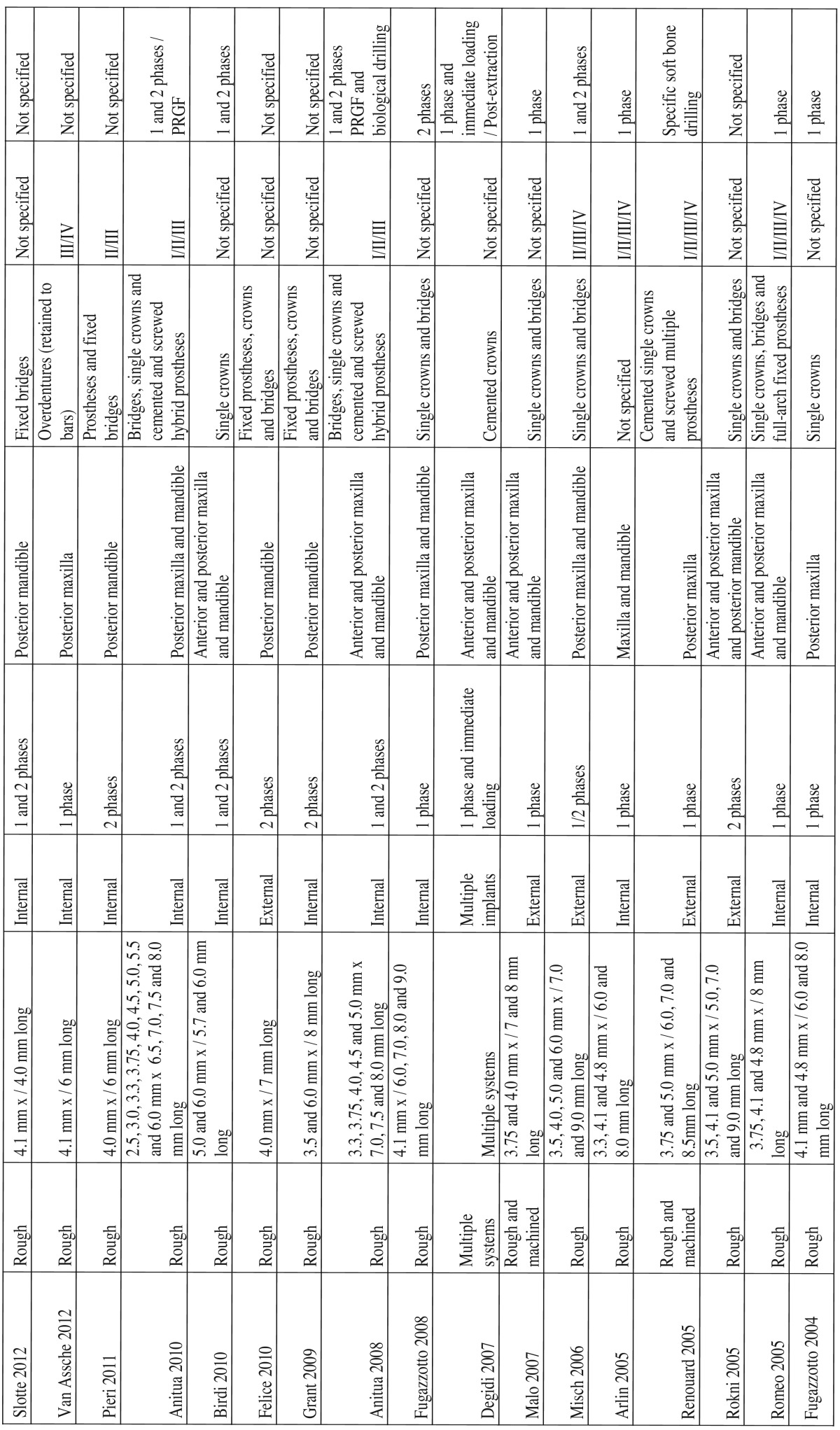
Variables associated to treatment characteristics.

The studies included in this review used implants with a wide variety of designs and surface treatments. The length of the implants ranged between 4.0-9.0 mm, while the implant diameter ranged between 2.5-6.0 mm. All the studies presented results corresponding to implants with rough surfaces subjected to different treatments. Five of the 37 studies presented results comparing short implants with a rough surface versus short implants with a machined surface.

Twenty-eight of the 37 studies presented results on implants with internal connection, while only 7 studies published results on implants with external connection. One study, published by Mendoça *et al.* (10), compared short implants with both internal and external connection. Another two studies, published by Sánchez-Garcés (11) and Degidi (12), employed multiple implant systems, without offering further information on the type of connection involved.

In relation to the treated maxilla, 24 studies presented results on implants placed both in the maxilla and in the mandible. 9 studies presented results only in the mandible, while only 5 studies presented results on implants placed only in the maxilla.

Regarding implant location, 23 of the studies published results on implants placed in the posterior regions of both maxillae. Another 10 studies published results on implants placed both in the anterior and the posterior areas while four articles failed to specify implant location.

Regarding the characteristics of the surgical technique, all of the reviewed studies raised full-thickness flaps for implant placement. Seventeen articles provided results on short implants placed using two-step surgery, while 14 studies performed single-step surgery. Only Anitua *et al.* (13) and Degidi *et al.* (12) presented results with an immediate loading approach. Six studies included implants placed in both single and two-step surgical procedures.

Two studies modified the drilling protocol. These two studies adapted the surgical technique to increase implant stability in cases of soft bone. Another two studies, used surgical templates to guide the drilling of the implants.

The implants included in this review were used to support different types of prosthetic restorations such as fixed prostheses (single or multiple) and over dentures (with splinted implants). Twenty-four studies presented clinical results with short implants supporting single restorations, though only 5 of them published data on short implants supporting single-unit crowns on an exclusive basis. On the other hand, 22 studies included clinical results on short implants splinted with fixed prostheses to other implants of the same or greater length.

Ten of the reviewed studies presented information on the insertion torque applied at the time of implant placement. The values ranged from a minimum of ≤ 15 N in the study of Rossi *et al.* (14), to a maximum of 60 N in the studies published by Anitua *et al.* (15) and Pieri *et al.* (16).

Sixteen studies recorded information on the bone quality of the areas in which the short implants were placed. Fourteen of these articles recorded short implant placement in type III and type IV bone.

3. Variables associated to treatment outcome ( Table 3).

**Table 3:**
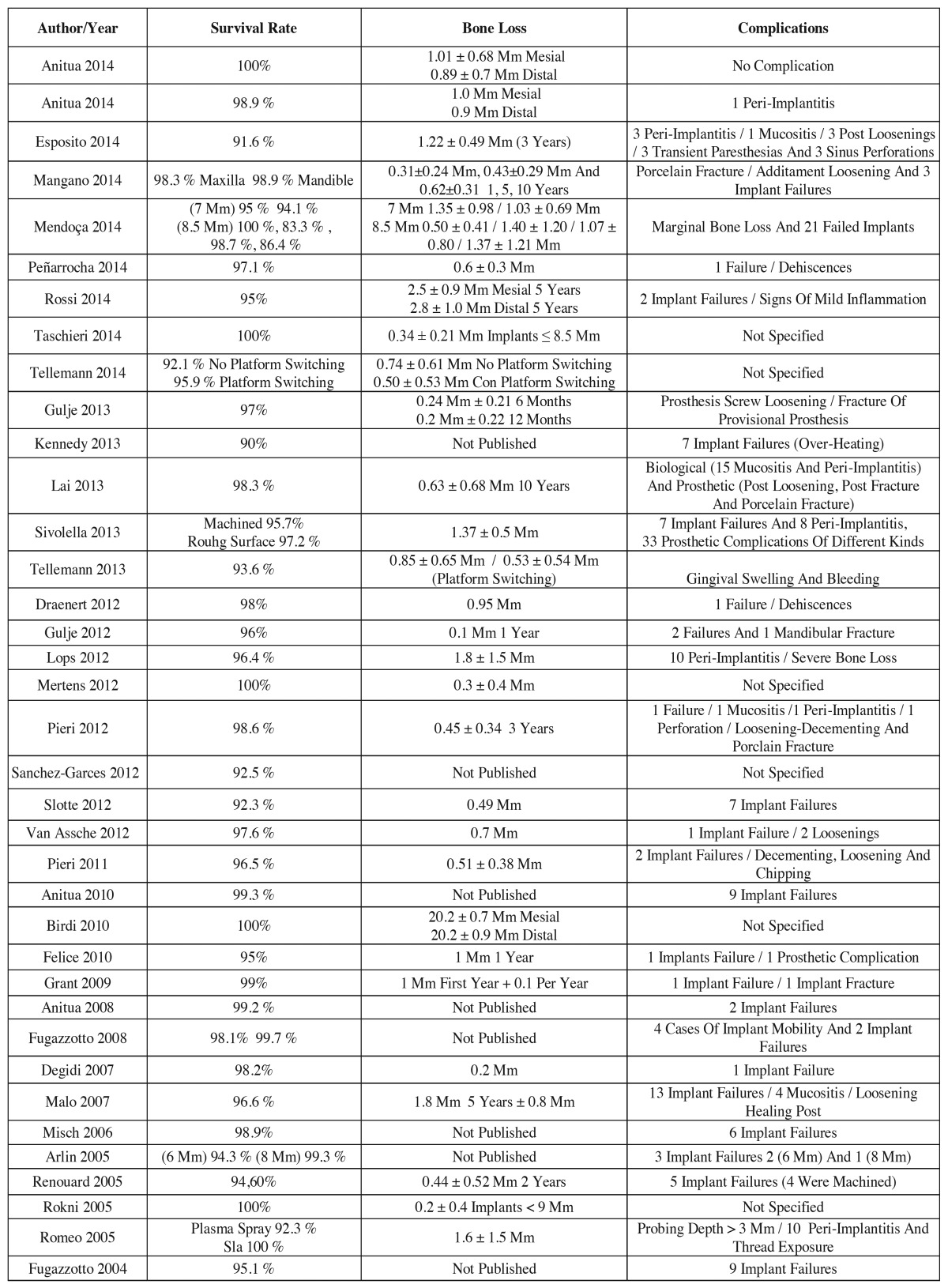
Variables associated to treatment outcome.

The implant survival rates ranged from 83.3% referred to 6 implants measuring 8.5 mm in length and placed in the upper maxilla in the study of Mendoça *et al.* (10) to 100% reported in the studies of Anitua *et al.* (4), Taschieri *et al.* (6), Mertens *et al.* (17), Birdi *et al.* (18) and Rokni & Todescan (19).

Twenty-nine studies measured the changes in peri-implant bone level after implant loading. The bone loss around the implants ranged from 0.1 mm after one year in the study published by Gulje *et al.* (20) to 2.5 ± 0.9 mm / 2.8 ± 1.0 mm measured after 5 years in the study of Rossi *et al.* (14).

A total of 31 studies provided information on the complications associated with short implants. A number of problems related to implant placement were recorded, such as implant loss (135 implants in 23 studies), mucositis and peri-implantitis (51 implants in 8 studies), mobility of the implant (4 implants in 1 study), perforation of the sinus membrane (4 perforations in 2 studies), and mandibular fracture (1 fracture). Other complications recorded in the studies were related to the prosthesis, including cement loss, loosening, or fracture of the prosthesis or of some of its components (screw or abutment), and fracture of the implant (1 case).

## Discussion

Short implants (< 10 mm in length) produce results comparable to those obtained with implants of greater length after prolonged follow-up periods, as reported by Monje *et al.* in their meta-analysis published in 2013 (21). Our review included only 6 randomized clinical trials supporting this affirmation. The minimum duration of follow-up was 12 months in all the studies, thus allowing us to conduct an analysis of the middle-term results obtained. The patient sample was quite large and included individuals 

who were partially or totally edentulous in both maxillae. Due to the great variety of the implants analyzed,

it is difficult to establish a relationship between the different implant surface characteristics, diameters and lengths and the implant survival.

We found most of the reviewed studies to publish survival rates over 95%. These are high percentages, as seen for example in the studies published by Anitua *et al.* (22), Lops *et al.* (23) and Romeo *et al.* (9). All three studies involved a follow-up period of over 10 years, with survival rates greater than those recorded for implants placed in posterior regions of the upper maxilla using the sinus lift with lateral window technique, according to a recent systematic review published by Del Fabbro *et al.* (24). These authors recorded a survival rate of about 93.7% for implants placed in grafted bone.

Likewise, in relation to the treatment of atrophic mandibles, Al-Nawas *et al.* (25), in their systematic review, published survival results in the order of 96% for implants placed in grafted bone using different techniques. It therefore can be affirmed that short implants offer good clinical results with shorter treatment times, low morbidity rates, and few intraoperative complications.

As seen from our review, another factor to be taken into account is the type of implant surface involved. The survival results obtained are much better for implants with a rough surface than for implants with a machined smooth surface. Furthermore, in the case of shorter implants and narrow-diameter implants, where the bone-implant contact surface area is reduced, it is essential for the surface treatment to provide a correct osseointegration. On the other hand, as indicated by Heitz-Mayfield & Mombelli in their systematic review (26), it is also true that surface roughness is associated to an increased risk of peri-implantitis if good maintenance is not ensured. In our review, this circumstance, together with implant loss, was the most common biological complication.

In the three-dimensional study of finite elements published by Petrie & Williams (27), low biomechanical stress levels were associated to large-diameter implants. Increasing the diameter was found to result in a 3.5-fold decrease in crestal strain. In contrast, an increase in implant length only resulted in a 1.65-fold decrease in crestal strain. This author considered implant diameter to have a stronger influence than implant length - in agreement with other authors such as Anitua *et al.* (28).

Most of the studies reported results on implants placed in both maxillae. The few studies presenting data on short implants exclusively placed in the upper maxilla also described good results. According to the systematic review published by Srinivasan & Vazquez (29) also published survival rates between 92.2% and 100% for short implants measuring 4-7.5 mm in length - with a higher failure rate in the upper maxilla. In the mentioned study, 297 implants were placed in the upper maxilla. 13 of this 297 implants were seen to fail. In the mandible 826 implants were placed and only 19 out of this 826 implants failed. These differences can be explained by the fact that the posterior region of the upper maxilla is characterized by type IV bone. In this regard, the presence of poorer quality bone is a decisive factor in quantifying implant survival.

Another of the objectives of our review was to analyze peri-implant bone loss. According to the results obtained, such loss does not seem to be influenced by implant length. This is consistent with the findings of the systematic review published by Monje *et al.* (30).

These authors found no statistically significant differences in bone loss between standard-length implants versus shorter implants. In this respect, the new implant designs and types of connections appear to play a very important role. More rigid internal connections with fewer micromovements cause the peri-implant tissues to remain more stable over time. In this regard, mention can be made of the study published by Mendonça *et al.* (10), in which the poorest results were obtained with non-splinted externally connected implants presenting a smooth machined surface. Most of the studies in our review used internal connections.

Another important parameter analyzed in our review is whether or not prosthetic splinting of short implants is necessary. In this regard, a number of authors such as Misch & Steigenga (31) recommend the splinting of short implants.

As an example, the retrospective study published by Misch & Steigenga combined the splinting of short implants with implants of standard size (62 implants). At the same time, splinting of multiple short implants was also carried out (174 implants). On the other hand, in the same study 64 short implants were placed in the mandible and 38 in the upper maxilla supporting unit restorations. The success rate was higher for splinted short implants. On examining the different studies included in our review, most of them were seen to use splinted prostheses. However, many of the publications also used short implants to support single crowns, with similar results.

Likewise in relation to the prosthetic rehabilitation of short implants, a disproportionate crown-implant ratio has not been identified as a decisive factor in treatment outcome. This is consistent with the observations of Birdi *et al.* (18), though other investigators argue that disproportion between the size of the crown and of the implant is indeed associated to a greater risk of fracture and loosening of the prosthesis. No authors *et al.* (32) found that despite the greater risk of loosening, the peri-implant bone levels are not significantly affected as a result. This implies that when using short implants to support single-unit restorations, loosening of the prosthesis is the main prosthetic complication.

## Conclusions

Despite the limitations inherent to this systematic review, the results obtained appear to confirm that short dental implants offer clinical results in terms of survival, bone loss and complications similar to those of longer implants. Further studies are needed, involving longer periods of follow-up, in order to confirm these conclusions.
